# A Simple, Efficient, Eco-Friendly Sample Preparation Procedure for the Simultaneous Determination of Hormones in Meat and Fish Products by Gas Chromatography—Mass Spectrometry

**DOI:** 10.3390/foods11193095

**Published:** 2022-10-05

**Authors:** Safae Chafi, Evaristo Ballesteros

**Affiliations:** Department of Physical and Analytical Chemistry, E.P.S of Linares, University of Jaén, Avenida de la Universidad, s/n, 23700 Linares, Spain

**Keywords:** hormones, meat, fish, solid-phase extraction, gas chromatography–mass spectrometry

## Abstract

Food safety can be severely compromised by the presence of chemical contaminants. This has raised a pressing need to develop efficient analytical methods for their determination at very low levels in complex food matrices. In this manuscript, we developed a simple, sensitive, fast, green analytical method for the determination of thirteen natural and synthetic hormones from different families including progestogens, estrogens and androgens in meat and fish products. The method involves direct extraction with a (9:1) acetonitrile–water mixture and subsequent purification of the extract by semi-automated solid-phase extraction on a sorbent column (hydrophilic–lipophilic copolymer of N-vinylpyrrolidone and divinylbenzene). This treatment enriches samples with the target compounds while removing proteins, lipids and other potential interferences from their matrix for the accurate determination of the analytes by gas chromatography–mass spectrometry, all within 15 min. The proposed method exhibits good linearity (*r* ≥ 0.996), low limits of detection (0.4–15 ng/kg), acceptable recoveries (90–105%) and relative standard deviations (≤7%); in addition, it is scarcely subject to matrix effects (1–20%). The method was successfully used to determine natural and synthetic hormones in meat and fish products from Spain, Portugal, Italy, Germany, Greece, Norway, Morocco and the USA. The analytes were found at especially high levels (30–1900 ng/kg) in mussels, beef and pork.

## 1. Introduction

In the last few decades, a number of naturally occurring and synthetic hormones referred to as “endocrine disrupting compounds” (EDCs) have aroused much interest as a result of their potential deleterious effects on human and animal health (carcinogenicity and endocrine disruption included) and on the environment [1,2]. The International Agency for Research on Cancer has deemed diethylstilbestrol and estrogens carcinogenic (group 1A), androgenic anabolic steroids probably carcinogenic (group 2A) and progestogens (group 2B) possibly carcinogenic to humans [3]. Food consumption is a major source of exposure to these natural and synthetic hormones, which are widely used to hasten industrial production in animal husbandry and aquaculture. The European Union Scientific Committee on Veterinary Measures relating to Public Health (SCVPH) has found that their use of bovine meat and meat production caused serious health problems [4]. Fish species are especially useful for biomonitoring water quality by virtue of their tendency to accumulate substantial amounts of endocrine-disrupting compounds (EDCs) through their gills, oral intake and surface skin exposure, all of which increase human health risks from fish consumption [5,6].

The illegal use of hormones such as estrogens, progestogens or androgens on animals by oral administration in feed/drinking water, injection or implantation can have unwanted results such as the presence of residual amounts of these substances in products of animal origin such as milk, meat and liver [7,8,9,10]. Several studies have shown the presence in fish tissues of estrogens, androgens and progestogens from wastewater treatment plant effluents, reservoirs, rivers, lakes, ponds and aquaculture tanks [6,11,12,13]. In response, the European Community (EC) has banned the use of substances with hormonal or thyrostatic action and β-agonists in livestock breeding and aquaculture [14]. In addition, the Joint FAO/WHO Expert Committee on Food Additives (JECFA) and the Codex Alimentarius Commission have established an acceptable daily intake of 0–0.05 μg/kg (body weight/day) for 17β-estradiol, 0–30 µg/kg bw for progesterone and 0–2 µg/kg bw for testosterone [15,16]. Complying with these regulations requires careful control of these compounds in edible animal tissues and foods of animal origin in EU countries and in countries exporting food to Europe under the EU directive 96/23/EC [17]. This goal is realized according to the national plan of each individual member state. As no maximum residue limits (MRLs) for the previous substances have been established, the Community Reference Laboratories (CRLs) have set a recommended range of concentrations (0.1–10 μg/L or µg/kg) to improve and harmonize the performance of analytical methods for determining hormonal growth promoters [18].

Gas chromatography (GC) in combination with mass spectrometry (MS) allow hormones in edible animal tissues to be easily determined with excellent sensitivity and selectivity thanks to the high separation efficiency of the former technique and reproducibility of fragmentation patterns in electron ionization (EI) mass spectra. As can be seen in Table S1 (Supplementary Materials), GC–MS has been used to analyze various types of meat and fish products including kidney fat, meat, milk, butter, egg and fish with acceptable sensitivity, accuracy and precision [6,19,20,21,22]. Although silylation is widely favored for derivatization prior to GC–EI-MS analysis [6,19,21,22,23], some prefer high performance liquid chromatography (HPLC) for determining hormones in animal tissues. For instance, ultra-high performance liquid chromatography coupled with Orbitrap high resolution mass spectrometry (UHPLC-Orbitrap-HRMS) has been successfully used to determine steroid hormones in meat [24,25]. As can be seen in Table S1, UHPLC or HPLC in combination with tandem mass spectrometry has also been used to determine hormones in meat and fish products [5,10,11,13,26,27,28,29,30].

Meat and fish are complex food matrices owing to the presence of a variety of compounds and the tendency of proteins and lipids to form suspensions. As a result, quantifying hormones at low (μg/kg to ng/kg) levels typically require a prior treatment such as protein precipitation, centrifugation, preconcentration and/or purification. Residual hormones present in animal tissue samples are usually subjected to extraction and clean-up with various techniques such as liquid–liquid extraction [19,24,27], accelerated solvent extraction [22], microwave-assisted extraction [11,20], focused ultrasound solid–liquid [5], ultrasound-assisted extraction [19,26], matrix solid-phase dispersive extraction [1,5,29] or QuEChERS (Quick, Easy, Cheap, Effective, Rugged and Safe method) [13,25,30]. In addition, clean-up by solid-phase extraction (SPE) has proved successful to suppress matrix interferences [11,19,20,22,26,28,31]. Applying the treatment via a flow system dramatically increases throughput and recoveries while lowering analytical costs through reduced reagent consumption [23].

This work was undertaken with a threefold purpose, namely: (1) to develop an especially sensitive, cost-effective green method for the determination of natural and synthetic hormones including progestogens, estrogens and androgens in edible animal tissues by gas chromatography–mass spectrometry in the electron impact mode (EI) with a run time of only 15 min and using the minimum volume of organic solvent; (2) to improve the efficiency of the sample preparation procedure by optimizing specific variables to avoid coextractive interferences from proteins, lipids and other interferents for optimal sensitivity and selectivity; and (3) to validate the ensuing method and use it to analyze the target analytes in real samples.

## 2. Materials and Methods

### 2.1. Chemicals and Reagents

High purity (over 99%) analytical standards were used throughout. The hormones studied were all purchased from Sigma–Aldrich (Madrid, Spain). Chromatographic grade solvents (acetone, propanol, ethanol, methanol, acetonitrile, diethyl ether, ethyl acetate, *n*-hexane and petroleum ether), triphenyl phosphate and derivatizing reagents (trimethylchlorosilane (TMCS) and *N*,*O*-*bis*(trimethylsilyl)trifluoroacetamide (BSTFA)) were obtained from Fluka (Madrid, Spain). HCl (reagent grade, 37%), NaCl and NaOH were purchased from Fluka. The sorbent Oasis-HLB (hydrophilic–lipophilic copolymer of *N*-vinylpyrrolidone and divinylbenzene, particle size 50–65 µm) was supplied by Waters (Madrid, Spain). Ultrapure water was obtained from a Milli-Q apparatus from Millipore (Bedford, MA, USA). All products were handled with care, using efficiently ventilated hoods, wearing protective gloves and avoiding inhalation or skin contact because hormones are usually toxic.

Standard stock solutions containing a 5 g/L concentration of the hormones 17α-ethinyl estradiol, 17β-estradiol, estriol, diethylstilbestrol, hexestrol, norethindrone, dihydrotestosterone, testosterone, progesterone, pregnenolone, androstenedione and others with identical concentration of levonorgestrel and estrone were dissolved in acetone to avoid crystallization. All standard solutions were stored in glass-stoppered bottles at 4 °C in the dark. Working-strength solutions were made on a daily basis by diluting an appropriate volume of stock solution in ultrapure water. The eluent was acetone containing 100 µg/L triphenyl phosphate as internal standard (IS) and prepared on a daily basis.

### 2.2. Equipment

The GC–MS system consisted of a FOCUS gas chromatograph interfaced to a DSQ II mass spectrometer equipped with an AI/AS 3000 autosampler and controlled by a computer running the software XCalibur from Thermo Electron S.A. (Madrid, Spain). Hormones were separated on a DB-5 fused silica capillary column (30 m, 0.25 mm i.d., 0.25 µm film thickness) coated with 5% phenylmethyl polysiloxane from Supelco (Madrid, Spain). Helium (purity 6.0) at a constant flow rate of 1 mL/min was used as carrier gas. The oven temperature program was as follows: 130 °C for 0.5 min, 40 °C/min ramp to 240 °C and 5 °C/min ramp to 280 °C, the last being held for 3.75 min. All analytes were successfully separated within 15 min. The transfer line and ion source temperature were 280, and 200 °C, respectively. The injection port temperature was 280 °C, and solvent delay was set at 5 min. Quantification was performed in the selected ion monitoring (SIM) mode, using an ionization energy of 70 eV. Table 1 lists the *m*/*z* values for the analytes. Before each injection in the splitless mode, the 5 μL syringe was rinsed with 10 μL of methanol and 3 μL of sample solution.

### 2.3. Sampling

Twelve different types of fish sample (hake, sea bass, sea bream, cod, turbot, croaker, salmon, anchovy, squid rings, prawns, shrimp and mussels) and eight of meat (chicken breast, lamb, beef hamburger, pork hamburger, turkey hamburger, chicken sausages, turkey sausages and pork loin) were purchased from fish stores, meat shops and supermarkets in six European countries (Spain, Norway, Italy, Greece, Portugal and Germany) in addition to Morocco and the USA. All samples were stored in sealed polypropylene bottles at −20 °C that were previously cleaned with methanol and dried out.

### 2.4. Sample Preparation

**Figure 1:**
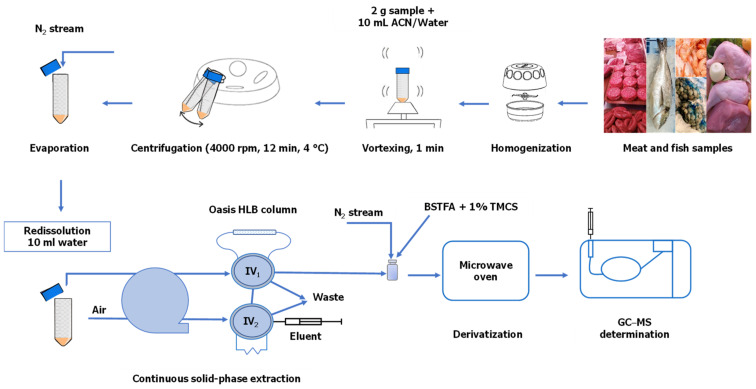
Experimental set-up for the preconcentration, derivatization and determination of natural and synthetic hormones in meat and fish products.

Figure 1 depicts the sample preparation procedure which involved the following steps: (1) homogenization of tissue samples (fish or meat) in a kitchen blender grinder (A320R1) from Moulinex (Barcelona, Spain) for storage at −20 °C until analysis; (2) weighing of 2 g in a 50 mL round polypropylene centrifuge tube for extraction with 10 mL of (9:1) acetonitrile–water mixture; (3) vortexing of the tube in a mixer from REAX Control (Heidolph, Kelheim, Germany) for 1 min and centrifugation at 4000 rpm (1720× *g*) at 4 °C on a Centrofriger BL-II model from JP Selecta (Barcelona, Spain) for 12 min; and (4) collection of the upper acetonitrile–water layer in another tube and careful evaporation under a nitrogen stream for redissolution in 10 mL of ultrapure water at pH 7 (adjusted with dilute NaOH or HCl).

Pretreated samples were subjected to continuous solid-phase extraction for preconcentration and purification, and eluted analytes were derivatized prior to GC–MS analysis. The continuous flow system used to preconcentrate and clean up hormones extracted from edible animal tissues was constructed from a Minipuls-3 peristaltic pump (Gilson, Villiers-le-Bel, France) fitted with poly(vinyl chloride) pumping tubes and two 5041 injection valves from Rheodyne (Cotati, CA, USA). The sorbent column was made from poly(tetrafluoroethylene) capillaries (3 mm i.d.), and end-caps were formed by fitting PTFE tubing (30 mm × 0.5 mm i.d.) into a 10 mm × 1 mm i.d. PTFE tube to facilitate insertion into the continuous system, with both ends being sealed with small swabs of cotton wool to prevent material losses [23].

The continuous SPE/derivatization procedure consists of several steps. In the preconcentration step, pretreated samples were aspirated at 5 mL/min through a sorbent column packed with 80 mg of Oasis-HLB and placed in the loop of injection valve IV_1_. Analytes were thus immediately retained onto the sorbent surface as the sample matrix was sent to waste. Then, any residual water was removed from the column by running an air stream at 5 mL/min, while the loop of valve IV_2_ (600 µL) was simultaneously filled with eluent (acetone containing 100 µg/L triphenyl phosphate as internal standard) by means of a syringe. This was followed by elution, where valve IV_s_ was switched to pass the loop contents (600 µL of eluent) through the column upstream to the sample to elute the analytes. The organic extract was collected in 0.5 mL sealed glass vials and carefully evaporated to dryness under a nitrogen stream. In the derivatization step, the dry residue was derivatized using 70 µL of BSTFA containing 1% of TMCS (derivatizing agent) and 35 µL of petroleum ether in a household microwave oven at 200 W for 4 min. Finally, aliquots of 1 µL were injected into the GC–MS equipment, operating in the SIM mode, to determine the silylated derivatives, with the exception of progesterone and androstenedione as they did not undergo derivatization. The sorbent column was conditioned by passing 1 mL of methanol, 1 mL of acetone and 2 mL of Milli-Q water in this sequence. The sorbent column remained serviceable for about 2 months.

## 3. Results and Discussion

### 3.1. Optimization of Sample Treatment

The success of an analytical method for determining analytes at trace levels (ng/kg to µg/kg) in complex matrices such as meat and fish depends largely on the efficiency of the sample preparation procedure. A suitable sample treatment should afford (a) isolation of the analytes from the sample, (b) removal of matrix interferents (proteins, lipids and other compounds) and (c) protection of the analytical equipment. Meeting these requirements entails carefully optimizing the variables most markedly affecting the analytical outcome.

**Figure 2:**
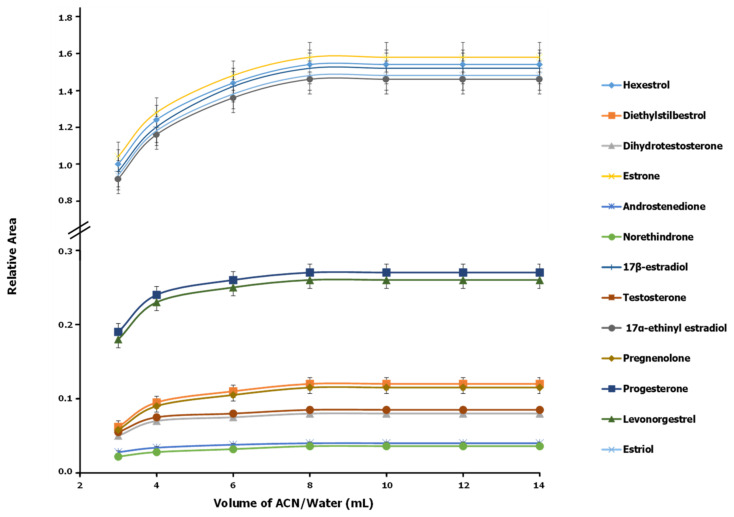
Influence of the volume of 9:1 *v*/*v* acetonitrile–water on the extraction of natural and synthetic hormones from meat (chicken breast) (*n* = 3).

An appropriate choice of extraction solvent is essential to ensure the acceptable recovery of analytes while removing proteins and lipids to avoid interferences. Methanol is the most frequently used organic solvent for extracting hormones from animal tissues [9,11,19,20,24,27,32]. However, acetonitrile is often preferred because lower lipids are less soluble in it, and the resulting extracts are cleaner, which prevents the blocking of the SPE system [6,7,10,26,30,33]. In this work, we assessed methanol, acetonitrile, diethyl ether, ethanol, propanol, acetone and mixtures thereof (acetonitrile–water, methanol–water, acetonitrile–methanol and acetonitrile–ethyl acetate in proportions from 9:1 to 1:9 *v*/*v*) for their extraction efficiency. A portion of 2 g of blank meat or fish tissue was spiked in triplicate with the analytes at a 500 ng/kg concentration. Then, a volume of 10 mL of each individual or mixed solvent was added, and the mixture was centrifuged at 5000 rpm at 4 °C for 10 min. A 9:1 *v*/*v* acetonitrile–water mixture was demonstrated to be the best solvent; it disrupted protein–hormone binding interactions and afforded quantitative extraction of all analytes as a result. Aqueous acetonitrile provided higher recoveries and a more efficient precipitation of proteins than alternative solvents [23,31,34]. The influence of the volume of 9:1 *v*/*v* acetonitrile–water mixture used was examined over the range 3–14 mL. As can be seen from Figure 2, peak areas increased with increasing volume from 3 to 8 mL; in addition, above 8 mL extraction was quantitative for all analytes, and no protein remained in the extracts after centrifugation because the protein fraction had precipitated. A volume of 10 mL was thus selected for further testing. Centrifugation-related variables are especially influential on the efficiency with which lipids and precipitated animal tissue proteins can be removed from extracts at low temperatures. In this work, the effects of the centrifugation speed, time and temperature were evaluated. The ranges studied for these variables were 1000–5000 rpm (430–2150× *g*), 5–16 min and 2–25 °C, respectively. As can be seen in Table S2 (Supplementary Materials), the optimum values for the three variables were 4000 rpm (1720× *g*), 12 min and 4 °C, respectively. The optimization results for all variables were similar for the fish and meat samples.

After that, we performed purification of the extract and concentration of the analytes using a continuous SPE method previously developed by our group to quantify synthetic and natural hormones in environmental water samples [35]. In order to conform with the principles of green chemistry, these parameters were evaluated: suitable sorbent material, effective eluent, minimum amount of sorbent and minimum volume of eluent. In this way, the highest sorption efficiency (close to 100%) was obtained with 80 mg of Oasis-HLB. On the other hand, acetone was selected as the most efficient eluent for all analytes of the organic solvents tested. In addition, the optimum volume to elute all target compounds was 600 µL. Table S2 shows the optimal ranges and selected values for all the variables affecting the efficiency of the sample preparation, continuous SPE process and hormone derivatization.

**Figure 3:**
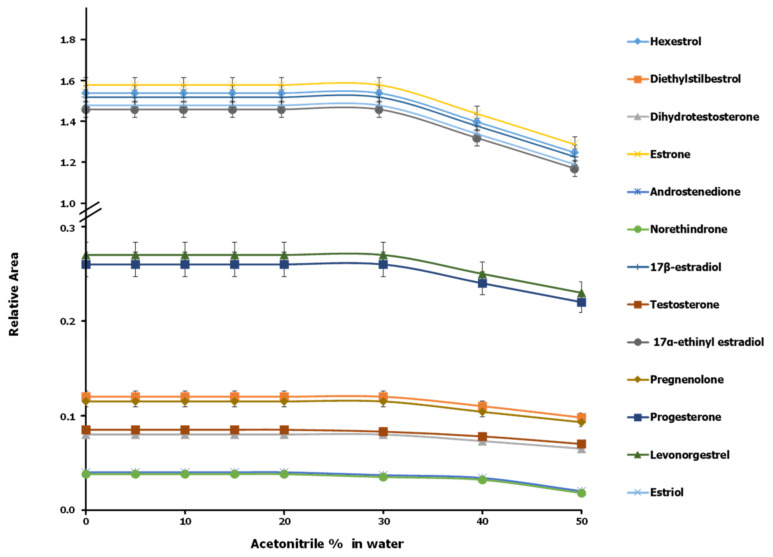
Influence of acetonitrile present in pretreated samples on the sorption efficiency of the continuous SPE system (*n* = 3).

The effect of the presence of acetonitrile in the pretreated samples on the sorption efficiency of the SPE system was evaluated using various aqueous standards containing a 500 ng/L concentration of each analyte in variable proportions of acetonitrile ranging from 0 to 50% *v*/*v* at pH~7. The solutions were aspirated through the sorbent column, packed with 80 mg of polymeric sorbent (Oasis-HLB). As can be seen from Figure 3, acetonitrile had no adverse effect on the retention of the hormones in proportions up to 20% for androgens and 30% for estrogens and progestogens. However, higher proportions of acetonitrile significantly detracted from hormone sorption. This was a result of too high a proportion of acetonitrile causing bonds to break and for hormones to be dissolved through partitioning between a polar phase (water) and a nonpolar one (Oasis-HLB) via hydrogen bonding and π–π interactions between the analytes and the underlying sorbent surface, which strongly reduced the adsorption of the analytes. This shortcoming was circumvented by evaporating the solvent present in the supernatant to a final volume of 1 mL under a stream of ultra-high-purity N_2_, redissolving to 10 mL and adjusting to pH~7.0.

### 3.2. Matrix Effects

Signal suppression or enhancement due to the effect of the sample treatment was assessed via the matrix effects. For this purpose, the slopes of the matrix-matched calibration curves for the hormones were compared with those of an external calibration graph constructed from standard solutions. The matrix effects were thus calculated using the following equation [36]:

ME = [(SCC_m_/SCC_s_) − 1] × 100 (1)

where SCC_m_ and SCC_s_ are the slope of the calibration curve in the matrix and solvent, respectively. The endogenous amount of the analytes in the samples was taken into account for the calculation of the ME, and the signal of this amount was subtracted from the signal of each point of the calibration curves. The matrix effects were classified as negligible [(0%)–(±10%)], mild [(±10%)–(±20%)], medium [(±20%)–(±50%)]) or strong (> ±50%) [37], with positive values denoting signal enhancement and negative values denoting suppression. As can be seen from Table 2, the matrix effects were negligible for most analytes in both types of matrix. In addition, more than 80% of the ME values were negligible (0% to ±10%), with the remaining 20% falling in the mild range (±11 to ± 20%). Therefore, the matrix effects never exceeded 20% suppression or enhancement, which is quite acceptable for a sample treatment.

### 3.3. Validation of the Proposed Method

The proposed SPE–GC–MS method was evaluated for its analytical performance in terms of its linearity, sensitivity, within- and between-day precision and recovery under the optimal extraction/clean-up conditions described in Section 2.4. A blank was inserted after each batch of three samples to prevent contamination from the analyst or the laboratory setting.

Linearity was assessed from the calibration graphs obtained by spiking 2 g of uncontaminated chicken breast or hake samples with the analytes at concentrations over the range 1.5–35,000 ng/kg. The linearity was good in all cases (correlation coefficients ranged from 0.996 to 0.999). Sensitivity was assessed as limits of detection (LODs), which were taken to be analyte concentrations giving chromatographic peaks equal to three times the standard deviation background noise divided by the slope of each calibration graph. The LODs ranged from 0.4 to 15 ng/kg (Table 1). Precision, as relative standard deviation (RSD) and recovery were assessed by analyzing in triplicate 11 uncontaminated meat (chicken) and fish (hake) samples spiked with target compounds at three different concentrations (100, 250 and 1500 ng/kg). Within-day precision ranged from 3.1% to 6.3% and between-day precision, as calculated on seven different days, from 3.4% to 6.8%. As most of the samples contained some analyte, the recoveries were calculated by subtracting the previously quantified endogenous compounds from their total content. The average recoveries of analytes added at three different concentration levels (100, 250 and 1500 ng/kg) to meat and fish samples ranged from 90% to 105% (Table 2). Therefore, the sample preparation method used affords the accurate determination of hormones in different types of fish and meat samples with little or no interference from the matrix.

### 3.4. Determination of Hormones in Meat and Fish Products

The practical use of the developed method was demonstrated by analyzing 20 different real-world fish and meat samples from eight different countries, namely: Spain, Portugal, Italy, the USA, Germany, Greece, Norway and Morocco. For this purpose, 2 g of each sample was analyzed in triplicate as described in Section 2.4 and followed by a blank. The results are summarized in Table 3.

All the fish samples except the hake, squid rings and shrimp contained at least one analyte. The mussels contained up to five (viz., dihydrotestosterone, progesterone, pregnenolone, estrone and 17β-estradiol) at concentrations from 0.091 to 1.9 µg/kg. These concentration levels are much lower than those found by Omar et al. (2018) in marine mollusks (up to 256 μg/kg), possibly as a result of the filtering of the large volumes of surface water used by mussels to feed and breathe, their long, sedentary life, their worldwide distribution and their low procurement costs [38]. Therefore, mussels can be effective bioindicators for the presence of chemical pollutants and useful in ecotoxicology and monitoring programs [39]. Norethindrone, estriol, 17α-ethinyl estradiol and testosterone were found in none of the fish samples, at least not at concentrations quantifiable with the developed method. Progesterone was present in three of the four maricultured fish samples (sea bass, turbot and croaker) at levels from 0.13 to 0.46 µg/kg, which are lower than those found by Ismail et al. (2021) in samples from Malaysia (0.80–9.78 µg/kg) [32]. Unlike the fish samples studied here which contained no diethylstilbestrol, this synthetic estrogen was previously found in various types of fish (catfish, eel fish and white bream) at concentrations from 2.20 to 15.60 µg/kg [6,13,29]. Other samples including cod, anchovies and salmon contained one or more of the following four hormones at concentrations from 0.020 to 0.64 µg/kg: estrone, dihydrotestosterone, progesterone and levonorgestrel.

As shown in Table 3, all meat products except the chicken breast from Portugal contained progesterone (0.16–0.78 μg/kg), but none contained the synthetic progestogens norethindrone and levonorgestrel. The highest concentrations of progesterone were those in the beef and pork hamburger samples (0.78 and 0.70 μg/kg, respectively). In any case, these levels are lower than those previously found by López-García et al. (2018) and Di Donna et al. (2015) in beef from Spain and Italy (1.70 and 1.9–5.4 μg/kg, respectively) [25,27]. Testosterone was detected in two samples (beef hamburger and pork hamburger), albeit at concentrations lower than the maximum levels recommended by CRL (2007): 0.03–0.20 μg/kg [18]. The estrone concentration of the chicken breast (0.072 µg/kg) is consistent with previously reported values (0.09–0.10 µg/kg) [30]. Neither of the three synthetic estrogens (hexestrol, diethylstilbestrol and 17α-ethinylestradiol) nor the natural estrogen (estriol) were found in any samples. The concentrations of 17β-estradiol in beef found here (0.17–0.55 µg/kg) are lower than those previously reported by Zhao et al. (2014) (0.26–2.51 µg/kg) [26]. Progesterone and androstenedione were present in the Turkish sausage, but only the former was in the chicken sausage (0.16–0.58 μg/kg). In general, the meat samples contained lower overall concentrations of hormones than the fish samples. Based on the results, the proposed method is an effective choice for determining hormones in real samples. By way of example, Figure S1 shows the chromatogram for the chicken breast sample spiked with all hormones and processed by the developed method.

## 4. Conclusions

The aim of this work was to develop a fast, sensitive, economical, eco-friendly method for the extraction and determination of trace levels of thirteen natural and synthetic hormones including estrogens, androgens and progestogens in a variety of meat and fish samples. The method includes a green sample preparation procedure using low volumes of organic solvents and semi-automated continuous clean-up to ensure good extraction efficiency and sensitivity. Thus, the volume of organic solvent used for the preparation of the sample in the proposed method is 10.6 mL, compared to those used by other authors (e.g., 72 mL [7], 70 mL [19], 80 mL [26,27] or 46 mL [32]). The methodology developed allows hormones to be extracted from high complex food matrices providing cleaner extracts for purification and preconcentration through a continuous SPE system and subsequent determination by GC–MS, all in a very short time (15 min). In addition, the use of a closed SPE system avoids the loss of analytes and reduces the risk of sample contamination from the laboratory or the analyst.

The proposed method can detect residual hormone levels ranging from 0.4 to 15 ng/kg in meat and fish samples, which compares favorably with those of existing methods (Table S1). For example, the MSPE-QuEChERS-UHPLC-MS/MS method of Xiong et al. (2020) showed LODs of 20–3000 ng/kg for meat samples [30], and the QuEChERS–GC–MS method of Zhou et al. (2019) showed LODs of 30–1950 ng/kg for estrogens in fish samples [6]. The proposed method provides near-quantitative recoveries (90–105%) and low RSDs (3.1–6.8%). In contrast, the recoveries ranges achieved in other methodologies were 52–107% [10], 20–130% [11] and 66–115% [26], for example.

The method was successfully used to determine natural and synthetic hormones in meat and fish samples from various countries. Most of the samples contained progesterone at concentrations from 0.091 to 0.780 µg/kg. The analytes were present at especially high levels (0.030–1.9 µg/kg) in mussels, beef and pork hamburgers. In any case, such levels are lower than those previously reported by other authors (Table S1). They are lower than the recommended maximum concentration levels for natural and synthetic hormones in muscle set in the guidance paper of CRL [18].

## Figures and Tables

**Table 1:** Retention time and analytical figures of merit of the proposed method SPE-GC-MS and mass values used for MS detection.

Compounds	Linear Range (ng/kg)	^a^ r	^b^ LOD (ng/kg)	^c^ Precision RSD (%)				^d^ t_R_	^e^*m*/*z*		
				Meat		Fish			M^+^	[M-15]^+^	Additional Ions
				Intra-day	Inter-day	Intra-day	Inter-day				
**Estrogens**											
Hexestrol	1.5–35,000	0.999	0.4	3.5	6.8	4.3	5.9	7.08	414	399	**207**, 179
Diethylstilbestrol	20–35,000	0.997	5.2	3.5	5.9	5.0	5.4	7.17	**412**	397	383, 217
Estrone	1.5–35,000	0.996	0.4	6.3	6.4	3.1	5.9	9.94	**342**	327	218, 257
17β-estradiol	1.5–35,000	0.998	0.4	6.0	6.1	3.4	5.3	10.27	**416**	401	285, 326
17α-ethinyl estradiol	1.5–35,000	0.996	0.4	4.3	5.4	6.0	6.5	11.46	440	**425**	232, 300
Estriol	1.5–35,000	0.996	0.4	4.5	3.4	4.9	5.7	12.44	**504**	489	147, 311
**Androgens**											
Testosterone	25–35,000	0.996	7.5	4.4	5.4	3.2	3.5	10.46	**360**	345	270, 226
Dihydrotestosterone	25–35,000	0.996	7.6	5.3	6.4	3.5	5.0	9.75	362	347	**129**, 272
Androstenedione	50–35,000	0.999	15	5.5	6.8	3.9	4.4	10.37	**286** ^g^	–	244, 148
**Progestogens**											
Progesterone	10–35,000	0.999	2.5	6.2	6.1	5.3	5.5	12.14	314 ^g^	–	**124**, 272
Norethindrone	50–35,000	0.997	15	6.2	6.3	5.7	6.3	10.41	370	355	**231**, 298
Levonorgestrel	10–35,000	0.998	2.6	6.3	6.8	4.8	6.5	11.60	384	369	**355**, 281
**Others**											
Pregnenolone	20–35,000	0.999	5.3	5.1	6.5	4.9	5.3	11.12	388	373	**129**, 298

^a^ r: correlation coefficient; ^b^ LOD, limit of detection; ^c^ RSD, relative standard deviation (*n* = 11) for 100 ng/kg (*p* < 0.05); ^d^ t_R_: retention time; ^e^ base peaks used for quantification are boldfaced; *m*/*z* for IS (triphenyl phosphate): 77, 170, 325, **326;** and **^g^** progesterone and androstenedione are determined as non-derivatize.

**Table 2:** Percent recoveries and matrix effect of hormones on the different types of fish and meat samples.

	Percent Recoveries (±SD, *n* = 3)/Matrix Effect (%)													
Hormones	Fish Sample								Meat Sample					
	Sea Bass	Salmon	Cod	Mussels	Hake	Shrimp	Squid Rings	Anchovies	Chicken Sausage	Beef Hamburger	Pork Loin	Chicken Breast	Turkey Hamburger	Lamb
Hexestrol	95 ± 6 ^a^ 10% ^b^	96 ± 5 7%	101 ± 6 8%	95 ± 4 8%	104 ± 5 10%	95 ± 4 10%	91 ± 4 −4%	94 ± 4 −6%	91 ± 5 13%	102 ± 4 12%	100 ± 6 −14%	102 ± 5 −12%	93 ± 6 −4%	103 ± 5 −16%
Diethylstilbestrol	96 ± 5 −6%	102 ± 5 3%	100 ± 5 11%	99 ± 6 −9%	103 ± 6 8%	93 ± 5 −2%	103 ± 6 6%	102 ± 6 11%	94 ± 6 18%	94 ± 4 5%	99 ± 4 11%	99 ± 4 −5%	95 ± 6 5%	94 ± 6 −19%
Estrone	103 ± 4 2%	97 ± 5 −9%	94 ± 6 −2%	90 ± 5 −5%	98 ± 5 −12%	98 ± 6 9%	102 ± 5 7%	97 ± 5 −4%	96 ± 5 −3%	100 ± 5 4%	92 ± 5 12%	91 ± 5 −14%	96 ± 5 5%	100 ± 4 4%
17β-estradiol	101 ± 5 −10%	96 ± 5 −3%	90 ± 6 4%	104 ± 6 −1%	99 ± 5 4%	101 ± 4 5%	99 ± 5 −4%	92 ± 6 −1%	101 ± 5 3%	92 ± 6 9%	97 ± 4 7%	102 ± 6 −3%	103 ± 6 3%	92 ± 6 8%
17α-ethinylestradiol	92 ± 4 4%	97 ± 4 10%	94 ± 6 10%	91 ± 5 4%	94 ± 4 −10%	100 ± 5 5%	103 ± 6 −8%	105 ± 4 −8%	102 ± 5 −11%	92 ± 4 −7%	101 ± 6 6%	101 ± 4 −7%	99 ± 5 2%	92 ± 7 −17%
Estriol	96 ± 4 −8%	100 ± 5 6%	99 ± 4 6%	100 ± 6 −10%	99 ± 6 −6%	104 ± 6 4%	101 ± 5 3%	93 ± 5 −8%	101 ± 5 3%	97 ± 4 5%	93 ± 6 −9%	95 ± 5 −6%	94 ± 5 10%	103 ± 6 5%
Testosterone	100 ± 5 −13%	103 ± 6 −7%	102 ± 6 −14%	91 ± 4 −2%	100 ± 5 −1%	92 ± 4 −10%	97 ± 4 −14%	100 ± 4 2%	103 ± 6 1%	93 ± 6 −19%	96 ± 5 7%	94 ± 4 −10%	101 ± 6 −2%	96 ± 6 −5%
Dihydrotestosterone	100 ± 4 −12%	96 ± 4 −3%	104 ± 5 4%	94 ± 5 −7%	100 ± 6 2%	92 ± 6 −2%	93 ± 5 −10%	94 ± 6 −5%	102 ± 4 7%	98 ± 5 −17%	100 ± 4 −1%	101 ± 5 −8%	104 ± 6 −3%	95 ± 5 −7%
Androstenedione	102 ± 6 7%	97 ± 5 4%	92 ± 5 −11%	93 ± 5 3%	101 ± 5 −13%	101 ± 6 2%	94 ± 6 −4%	98 ± 5 4%	98 ± 4 −11%	99 ± 5 −13%	95 ± 6 2%	97 ± 6 −19%	97 ± 4 5%	105 ± 6 −6%
Progesterone	101 ± 6 −3%	99 ± 4 10%	93 ± 4 2%	100 ± 6 10%	94 ± 4 −5%	100 ± 6 −1%	93 ± 4 8%	103 ± 5 −13%	96 ± 6 −14%	96 ± 6 5%	93 ± 6 −3%	94 ± 4 3%	96 ± 6 2%	91 ± 4 −9%
Norethindrone	99 ± 7 −3%	103 ± 4 11%	97 ± 6 6%	92 ± 4 −5%	91 ± 6 6%	104 ± 5 −7%	98 ± 4 −10%	101 ± 6 −9%	94 ± 6 −2%	104 ± 6 −7%	97 ± 6 7%	101 ± 6 8%	90 ± 7 8%	95 ± 5 2%
Levonorgestrel	91 ± 4 9%	104 ± 6 4%	92 ± 5 −16%	93 ± 4 −8%	104 ± 4 3%	101 ± 5 −3%	93 ± 4 5%	98 ± 6 6%	101 ± 5 −9%	94 ± 4 −19%	93 ± 6 −18%	95 ± 6 8%	99 ± 4 8%	100 ± 4 12%
Pregnenolone	103 ± 5 3%	96 ± 6 10%	100 ± 5 3%	95 ± 6 −4%	99 ± 5 −11%	98 ± 6 −2%	92 ± 6 −5%	97 ± 5 −12%	91 ± 6 −3%	103 ± 6 5%	101 ± 4 −19%	102 ± 4 −9%	97 ± 4 2%	102 ± 5 −7%

^a^ Percent recoveries (% ±SD, *n* = 3) of hormones spiked in fish and meat samples (100 ng/kg); ^b^ matrix effects are expressed as the ratio between the calibration curve slope in the matrix and calibration curve slope in the solvent and are calculated using the following formula: MEs = [(calibration curve slope in matrix/calibration curve slope in solvent) − 1] × 100.

**Table 3:** Determination of natural and synthetic hormones in meat and fish samples from various markets around the world (mean values ± standard deviation, ng/kg, *n* = 3).

Compound ^a^		Hexestrol	Estrone	17β-Estradiol	Testosterone	Dihydrotestosterone	Androstenedione	Progesterone	Levonorgestrel	Pregnenolone
Fish sample	Sea bass (S)	nd ^b^	nd	nd	nd	nd	nd	130 ± 10	nd	nd
	Salmon (N)	nd	nd	nd	nd	nd	nd	640 ± 40	30 ± 2	nd
	Cod (N)	nd	60 ± 4	nd	nd	26 ± 2	nd	550 ± 30	20 ± 1	nd
	Mussels (I)	nd	97 ± 5	470 ± 30	nd	1900 ± 100	nd	91 ± 5	nd	810 ± 50
	Hake (S)	nd	nd	nd	nd	nd	nd	nd	nd	nd
	Anchovies (M)	nd	140 ± 10	nd	nd	nd	nd	nd	nd	nd
	Sea Bream (G)	20 ± 3	420 ± 20	nd	nd	nd	nd	nd	nd	nd
	Prawns (S)	nd	nd	nd	nd	nd	nd	190 ± 10	400 ± 20	nd
	Shrimp (M)	nd	nd	nd	nd	nd	nd	nd	nd	nd
	Turbot (S)	nd	nd	nd	nd	nd	nd	460 ± 30	nd	nd
	Croaker (S)	nd	nd	nd	nd	nd	nd	310 ± 20	nd	nd
	Squid rings (P)	nd	nd	nd	nd	nd	nd	nd	nd	nd
Meat sample	Chicken sausage (Ge)	nd	nd	nd	nd	nd	nd	160 ± 10	nd	nd
	Turkey sausage (S)	nd	nd	nd	nd	nd	320 ± 20	580 ± 30	nd	nd
	Beef hamburger (USA)	nd	70 ± 4	440 ± 30	30 ± 2	nd	160 ± 10	780 ± 50	nd	nd
	Pork hamburger (S)	nd	390 ± 20	170 ± 10	200 ± 10	nd	270 ± 20	700 ± 40	nd	630 ± 40
	Turkey hamburger (S)	nd	nd	nd	nd	nd	nd	410 ± 30	nd	380 ± 20
	Pork loin (S)	nd	410 ± 20	550 ± 30	nd	nd	nd	440 ± 30	nd	nd
	Chicken breast (S)	nd	72 ± 4	nd	nd	nd	nd	460 ± 30	nd	nd
	Chicken breast (P)	nd	nd	nd	nd	nd	nd	nd	nd	nd
	Lamb (S)	nd	970 ± 60	nd	nd	nd	nd	290 ± 20	nd	nd

^a^ S: Spain; N: Norway; I: Italy; G: Greece; P: Portugal; Ge: Germany; M: Morocco; USA: the United States of America; and ^b^ nd: not detected.

## Data Availability

Data are contained within the article or Supplementary Materials.

## Supplementary Materials

The following supporting information can be downloaded at https://www.mdpi.com/article/10.3390/foods11193095/s1: Table S1: Comparison of the proposed and alternative methods for determining natural and synthetic hormones in meat and fish products from around the world; Table S2: Variables influencing the sample pretreatment, solid-phase extraction/clean-up process and derivatization of hormones; and Figure S1: Typical chromatogram in the SIM mode for 1 g of chicken breast spiked with all hormones.

## References

[1] Fan Y.B., Yin Y.M., Jiang W.B., Chen Y.P., Yang J.W., Wu J., Xie M.X. (2014). Simultaneous determination of ten steroid hormones in animal origin food by matrix solid-phase dispersion and liquid chromatography-electrospray tandem mass spectrometry. Food Chem..

[2] Ronquillo M.G., Hernandez J.C.A. (2017). Antibiotic and synthetic growth promoters in animal diets: Review of impact and analytical methods. Food Control.

[3] International Agency for Research on Cancer (1987). Overall evaluations of carcinogenicity: An updating of IARC monographs, volumes 1 to 42. IARC Monographs on the Evaluation of the Carcinogenic Risk of Chemicals to Humans.

[4] SCVPH (Scientific Committee on Veterinary Measures Relating to Public Health) (2002). Review of Previous SCVPH Opinions of 30 April 1999 and 3 May 2000 on the Potential Risks to Human Health from Hormone Residues in Bovine Meat and Meat Products, Adopted on 10 April 2002.

[5] Ros O., Izaguirre J.K., Olivares M., Bizarro C., Ortiz-Zarragoitia M., Cajaraville M.P., Etxebarria N., Prieto A., Vallejo A. (2015). Determination of endocrine disrupting compounds and their metabolites in fish bile. Sci. Total Environ..

[6] Zhou X., Yang Z., Luo Z., Li H., Chen G. (2019). Endocrine disrupting chemicals in wild freshwater fishes: Species, tissues, sizes and human health risks. Environ. Pollut..

[7] Yang Y., Shao B., Zhang J., Wu Y., Duan H. (2009). Determination of the residues of 50 anabolic hormones in muscle, milk and liver by very-high-pressure liquid chromatography-electrospray ionization tandem mass spectrometry. J. Chromatogr. B.

[8] Hoga C.A., Almeida F.L., Reyes F.G.R. (2018). A review on the use of hormones in fish farming: Analytical methods to determine their residues. CyTA J. Food.

[9] Rocha D.G., Lana M.A.G., Augusti R., Faria A.F. (2019). Simultaneous identification and quantitation of 38 hormonally growth promoting agent residues in bovine muscle by a highly sensitive HPLC-MS/MS Method. Food Anal. Methods.

[10] Moussa F., Mokh S., Doumiati S., Barboni B., Bernabò N., Al Iskandarani M. (2020). LC-MS/MS method for the determination of hormones: Validation, application and health risk assessment in various bovine matrices. Food Chem. Toxicol..

[11] Guedes-Alonso R., Sosa-Ferrera Z., Santana-Rodríguez J.J. (2017). Determination of steroid hormones in fish tissues by microwave-assisted extraction coupled to ultra-high performance liquid chromatography tandem mass spectrometry. Food Chem..

[12] Liu S., Chen H., Xu X.R., Hao Q.W., Zhao J.L., Ying G.G. (2017). Three classes of steroids in typical freshwater aquaculture farms: Comparison to marine aquaculture farms. Sci. Total Environ..

[13] Luo Z., Lu J., Li H., Tu Y., Wan Y., Yang Z. (2018). Air-assisted liquid-liquid microextraction integrated with QuEChERS for determining endocrine-disrupting compounds in fish by high-performance liquid chromatography–tandem mass spectrometry. Food Chem..

[14] European Commission (2003). Directive 2003/74/EC of The Europe-An Parliament and of the Council of 22 September 2003 Amending Council Directive 96/22/EC Concerning the Prohibition on the Use in Stockfarming of Certain Substances Having a Hormonal or Thyrostatic Action and of Beta-Agonists. Off. J. Eur. Comm..

[15] Joint FAO/WHO Expert Committee on Food Additives (JECFA) (2000). Evaluation of Certain Veterinary Drug Residues in Food World Health Organization.

[16] Codex Alimentarius (2018). Index of Veterinary Drugs. Maximum Residue Limits (MRLs) and Risk Management Recommendations (RMRs) for Residues of Veterinary Drugs in Food CX/MRL 2-2018. https://www.fao.org/fao-who-codexalimentarius/codex-texts/dbs/vetdrugs/veterinary-drugs/en/.

[17] European Commission (1996). European Community Council Directive 96/23/EC of 29 April 1996 on measures to monitor certain substances and residues thereof in live animals and animal products and repealing Directives 85/358/EEC and 86/469/EEC and Decisions 89/187/EEC and 91/664/EEC. Off. J. Eur. Comm..

[18] CRL (Guidance Paper) (2007). CRLs View on State of the Art Analytical Methods for National Residue Control Plans. https://www.bvl.bund.de/SharedDocs/Downloads/07_Untersuchungen/EURL_Empfehlungen_Konzentrationsauswahl_Methodenvalierungen_EN.html?nn=11011448.

[19] Seo J., Kim H.Y., Chul B.C., Hong J. (2005). Simultaneous determination of anabolic steroids and synthetic hormones in meat by freezing-lipid filtration, solid-phase extraction and gas chromatography–mass spectrometry. J. Chromatogr. A.

[20] Dévier M.H., Labadie P., Togola A., Budzinski H. (2010). Simple methodology coupling microwave-assisted extraction to SPE/GC/MS for the analysis of natural steroids in biological tissues: Application to the monitoring of endogenous steroids in marine mussels *Mytilus* sp. Anal. Chim. Acta.

[21] Capriotti A.L., Cavaliere C., Colapicchioni V., Piovesana S., Samperi R., Laganà A. (2013). Analytical strategies based on chromatography–mass spectrometry for the determination of estrogen-mimicking compounds in food. J. Chromatogr. A.

[22] Wolecki D., Caban M., Pazdro K., Mulkiewicz E., Stepnowski P. (2019). Simultaneous determination of non-steroidal anti-inflammatory drugs and natural estrogens in the mussels Mytilus edulis trossulus. Talanta.

[23] Azzouz A., Souhail B., Ballesteros E. (2011). Determination of residual pharmaceuticals in edible animal tissues by continuous solid-phase extraction and gas chromatography–mass spectrometry. Talanta.

[24] Vanhaecke L., Van Meulebroek L., De Clercq N., Bussche J.V. (2013). High resolution orbitrap mass spectrometry in comparison with tandem mass spectrometry for confirmation of anabolic steroids in meat. Anal. Chim. Acta.

[25] López-García M., Romero-González R., Garrido Frenich A. (2018). Determination of steroid hormones and their metabolite in several types of meat samples by ultra high performance liquid chromatography—Orbitrap high resolution mass spectrometry. J. Chromatogr. A.

[26] Zhao C., Yue Z., Wu H., Lai F. (2014). Simultaneous determination of fourteen steroid hormone residues in beef samples by liquid chromatography-tandem mass spectrometry. Anal. Methods.

[27] Di Donna L., Benabdelkamel H., Taverna D., Indelicato S., Aiello D., Napoli A., Sindona G., Mazzotti F. (2015). Determination of ketosteroid hormones in meat by liquid chromatography tandem mass spectrometry and derivatization chemistry. Anal. Bioanal. Chem..

[28] Van Tricht F., Essers M., Groot M., Sterk S., Blokland M., van Ginkel L. (2018). A fast quantitative multi-analyte method for growth promoters in bovine meat using bead-disruption, 96-well SPE clean-up and narrow-bore UHPLC-MS/MS Analysis. Food Anal. Methods.

[29] Tang J., Wang J., Yuan L., Xiao Y., Wang S., Wang X. (2019). Dummy molecularly imprinted matrix solid-phase dispersion for selective extraction of seven estrogens in aquatic products. Food Anal. Methods.

[30] Xiong X., Li D., Du Z., Xiong C., Jiang H. (2020). Magnetic solid-phase extraction modified Quick, Easy, Cheap, Effective, Rugged and Safe method combined with pre-column derivatization and ultra-high performance liquid chromatography-tandem mass spectrometry for determination of estrogens and estrogen mimics in pork and chicken samples. J. Chromatogr. A.

[31] Zhang Y., Xue X., Su S., Guo Z., Wang J., Ding L., Liu Y., Zhu J. (2018). A multi-Class, multi-residue method for detection of veterinary drugs in multiple meat using a pass-through cleanup SPE technique and UPLC-MS/MS analysis. Food Anal. Methods.

[32] Ismail N.A.H., Aris A.Z., Wee S.Y., Nasir H.M., Razak M.R., Kamarulzaman N.H., Omar T.F.T. (2021). Occurrence and distribution of endocrine- disrupting chemicals in mariculture fish and the human health implications. Food Chem..

[33] Wu H., Li G., Liu S., Hu N., Geng D., Chen G., Sun Z., Zhao X., Xia L., You J. (2016). Monitoring the contents of six steroidal and phenolic endocrine disrupting chemicals in chicken, fish and aquaculture pond water samples using pre-column derivatization and dispersive liquid—Liquid microextraction with the aid of experimental design methodology. Food Chem..

[34] Wang H., Zhou X., Zhang Y., Chen H., Li G., Xu Y., Zhao Q., Song W., Jin H., Ding L. (2012). Dynamic microwave-assisted extraction coupled with salting-out liquid-liquid extraction for determination of steroid hormones in fish tissues. J. Agric. Food Chem..

[35] Chafi S., Ballesteros E. (2022). A sensitive, robust method for determining natural and synthetic hormones in surface and wastewaters by continuous solid-phase extraction–gas chromatography–mass spectrometry. Environ. Sci. Pollut. Res..

[36] Matuszewski B.K., Constanzer M.L., Chavez-Eng C.M. (2003). Strategies for the assessment of matrix effect in quantitative bioanalytical methods based on HPLC-MS/MS. Anal. Chem..

[37] Castilla-Fernández D., Moreno-González D., Bouza M., Saez-Gómez A., Ballesteros E., García-Reyes J.F., Molina-Díaz A. (2021). Assessment of a specific sample cleanup for the multiresidue determination of veterinary drugs and pesticides in salmon using liquid chromatography/tandem mass spectrometry. Food Control.

[38] Omar T.F.T., Aris A.Z., Yusoff F.M., Mustafa S. (2019). Occurrence and level of emerging organic contaminant in fish and mollusk from Klang River estuary, Malaysia and assessment on human health risk. Environ. Pollut..

[39] Chovanec A., Hofer R., Schiemer F., Markert B.A., Breuve A.M., Zechmeisster H.G. (2003). Fish as bioindicators. Bioindicators and Biomonitors.

