# Supplementary material for: A Simple, Efficient, Eco-Friendly Sample Preparation Procedure for the Simultaneous Determination of Hormones in Meat and Fish Products by Gas Chromatography—Mass Spectrometry
Source: Foods. 2022 Oct 5;11(19):3095. doi: 10.3390/foods11193095 (PMC9562678; doi:10.3390/foods11193095)
Supplement: Supplementary file 1 [file foods-11-03095-s001.zip › foods-1927699-supplementary.pdf]

Supplementary Material

A simple, efficient, eco-friendly sample preparation procedure for the simultaneous determination of hormones in meat and fish products by gas chromatography−mass spectrometry

Safae Chafi, Evaristo Ballesteros

Department of Physical and Analytical Chemistry, E.P.S of Linares, University of Jaén, 23700 Linares, Jaén, Spain

Table S1. Comparison of the proposed and alternative methods for determining natural and synthetic hormones in meat and fish products from around the world.[a]

Analytes	Samples	Countries	Pretreatment methods	Analytical techniques	Analytical features	Concentration in real samples	Reference
4-Androstene-3,17-dione, stanozolol, 17α-hydroxyprogesterone, prednisone, prednisolone, testosterone, hydrocortisone, megestrol acetate, medroxyprogesterone acetate, and progesterone	Chicken, pork, beef and sausages	China	MSPD	UPLC−MS/MS	LOD: 0.01−0.05 µg/kg<br>RSD: < 10%<br>Recovery: 77−99%	Hydrocortisone: 2.4−3.4 µg/kg<br>Progesterone: 2.1 µg/kg	[1]
17β-Estradiol, 17α-ethinyl estradiol, diethylstilbestrol and others EDCs	Fish liver, brain and muscle	Spain	FUSLE, MSPD	LC−MS/MS	LOD: 3−20 µg/kg<br>RSD: < 26%<br>Recovery: 62−132%	Diethylstilbestrol: 7−8 µg/kg	[5]
Estrone, 17α-ethinyl estradiol, diethylstilbestrol and others EDCs	Fish muscle, liver, gill and gonad	China	QuEChERS, derivatization (BSTFA−1% TMCS)	GC−MS	LOD : 0.03−1.95 µg/kg<br>RSD: 0.95−11.9 %<br>Recovery: 47−128%	Estrone: 7.0−308 µg/kg<br>17α-Ethinylestradiol: 5.4−27.3 µg/kg<br>Diethylstilbestrol: 4.79−15.6 µg/kg	[6]
Dienestrol, diethylstilbestrol, hexestrol, progesterone, 17α-methyl testosterone, epitestosterone, 6 propyl-2-thiouracile, testosterone, melengestrol acetate, β-estradiol-3-benzoate, 17β-estradiol, 17α-ethinyl estradiol and estriol	Bovine matrices: muscle, kidney, liver and bile	Lebanon	QuEChERS	LC−MS/MS	LOD: 0.13−0.86 µg/kg<br>RSD: < 23 %<br>Recovery: 52−107%	Progesterone: 0.11−11.7 µg/kg<br>Testosterone: 0.5−9.5 µg/kg<br>Epi-testosterone: 0.89−280 µg/kg	[10]
Diethylstilbestrol, estrone, 17β-estradiol, 17α-ethynylestradiol, estriol, levonorgestrel, norethisterone, megestrol acetate, progesterone, testosterone, boldenone, nandrolone, cortisone, prednisone and prednisolone	Fish: *Sphoeroides marmoratus* and *Boops boops*)	Spain	MAE, SPE	UHPLC−MS/MS	LOD: 0.14−49 µg/kg<br>RSD: < 20%<br>Recovery: 20−130%	Norethisterone: 500−2710µg/kg<br>Prednisolone: 90 µg/kg<br>Nandrolone: 70−520 µg/kg<br>Progesterone: 560−3950 µg/kg	[11]
Progesterone, norethindrone, medroxyprogesterone, levonorgestrel, testosterone, 19-nortestosterone, methyltestosterone , stanozolol, androstenedione, dihydrotestosterone, estrone, 17β-estradiol, 17α-ethinyl estradiol, dienestrol, hexestrol, diethylstilbestrol and others EDCs	Grass and silver carp, white bream and catfish	China	AALLME, QuEChERS	HPLC−MS/MS	LOD: 0.03−0.8 µg/kg<br>RSD: 1.1−14.5 %<br>Recovery: 78−119%	Diethylstilbestrol: 2.2−3.6 µg/kg	[13]
Estradiol, testosterone, progesterone, zeranol and diethylstilbestrol	Beef	South Korea	LLE, SPE, UAE, derivatization (MSTFA/ NH_4I/DTE)	GC−MS	LOD: 0.1−0.4 µg/kg<br>RSD: 5−16%<br>Recovery: 68−106%	Progesterone: 0.3−36.5 µg/kg<br>Testosterone: 0.2 µg/kg	[19]
Testosterone, androstenedione, 17β-estradiol, estrone, pregnenolone, progesterone, dihydroandrostenedione, and dihydrotestosterone	Aquatic mollusks	France	MAE, SPE Derivatization (MSTFA/mercaptoethanol/ NH_4I)	GC−MS	LOD: 0.1−0.4 µg/kg<br>RSD: < 16%<br>Recovery: 85−114%	Progesterone/pregnenolone: <0.4−8.9 µg/kg	[20]
Estrone, 17β-estradiol, estriol and anti-inflammatories.	Mussels: *Mytilus edulis trossulus*	Poland	ASE, SPE, derivatization (BSTFA−1% TMCS)	GC−MS	LOD: 1−7 µg/kg<br>RSD: 0.24−7.8%<br>Recovery: 80−118%	Estrone: 256 µg/kg	[22]
17β-Nortestosterone, fluoxymesterone, progesterone, estrone, estriol, 17α-estradiol, 17β-estradiol, 17α-hydroxyprogesterone, 17α-ethinyl estradiol, diethylstilbestrol, dienestrol, α-zearalanol, β-zearalanol, 17α-testosterone, 17β-testosterone, hexoestrol, norgestrel, acetoxyprogesterone, medroxyprogesterone acetate and methyltestosterone and others hormones	Bovine muscle tissue	Belgium	LLE, SPE	UHPLC−Orbitrap −HRMS<br>UHPLC−MS/MS	LOD: 0.04−2.50 µg/kg<br>RSD: < 20%<br>Recovery: 81−119%	−	[24]
Hydrocortisone, cortisone, progesterone, prednisone, prednisolone, testosterone, melengesterol acetate, hydrocortisone-21-acetate, cortisone-21-acetate, testosteronepropionate, 17α-methyltestosterone, 6α-methylprednisolone, 17α-hydroxyprogesterone and medroxyprogesterone	Meat (chicken, pork and beef)	Spain	QuEChERS	UHPLC−Orbitrap −HRMS	LOQ: 1−2 µg/kg<br>RSD: 1−20 %<br>Recovery: 70−103%	Hydrocortisone: 2.8 µg/kg<br>Progesterone, 1.7 µg/kg	[25]

Hydrocortisone, testosterone, dehydroepiandrosterone, epitestosterone, etiocholanolone, androsterone, progesterone, testosterone propionate, pregnanediol, pregnanetriol, estriol, 17β-estradiol, 17α-estradiol and estrone	Beef	China	UAE, SPE	HPLC−MS/MS	LOQ: 0.004−0.24 µg/kg<br>RSD: 2.2−10.2 %<br>Recovery: 66−115%	Hydrocortisone: 0.22−0.46 µg/kg<br>Progesterone: 0.22−1.00 µg/kg<br>17β-Estradiol : 0.26−2.51 µg/kg<br>Etiocholanolone: 1.21−2.01 µg/kg	[26]
Testosterone, progesterone, pregnenolone, cortisone, hydrocortisone, androsterone, trenbolone acetate and melengestrol acetate	Beef	Italy, Poland, Netherlands, France	LLE, SPE, derivatization (NH_2OCH_3-HCl)	UHPLC−MS/MS	LOD: 0.21−0.45 µg/kg<br>RSD: 3−13.8 %<br>Recovery: 90−110%	Testosterone: 3.2−16.0 µg/kg<br>Progesterone: 1.9−5.4 µg/kg<br>Androsterone 4.1 µg/kg<br>Pregnenolone: 3.7−8.2 µg/kg<br>Cortisone: 2.0−24.8 µg/kg<br>Hydrocortisone: 0.73−1.8 µg/kg<br>Trenbolone acetate: 2.95 µg/kg	[27]
Estrone, 17β-estradiol, estriol, 17α-ethinyl estradiol, dienestrol, diethylstilbestrol and hexestrol	Eel, grass carp, goldfish, shrimp	China	DMI-MSPD	HPLC−MS/MS	LOD: 1.6−2.8 µg/kg<br>RSD: 2−5%<br>Recovery: 89−103%	Diethylstilbestrol: 5.2 µg/kg	[29]
Estrone,17β-estradiol, estriol and diethylstilbestrol, 17α--ethinyl estradiol, dienestrol, hexestrol, 2-methoxyestradiol, estradiol benzoate and estradiol-17-valerate and bisphenol A	Pork and chicken meat	China	QuEChERS-MSPE	UHPLC−MS/MS	LOD: 0.02−3 µg/kg<br>RSD: 0.48−15.1%<br>Recovery: 81−115%	Estrone:0.09–0.10 µg/kg<br>Diethylstilbestrol:0.55−0.92 µg/kg	[30]
Testosterone, mengestrol, methyltestosterone and others veterinary drugs	Pork, beef, mutton, chicken, pork liver, lamb liver and chicken liver	China	LLE, SPE	UPLC−MS/MS	LOD: 0.1−10 µg/kg<br>RSD: < 7.8%<br>Recovery: 80−116%	−	[31]
Testosterone, progesterone, diethylstilbestrol and others EDCs	Maricultured fish	Malaysia	UAE, SPE	HPLC−MS/MS	LOD: 0.49−2.08 µg/kg<br>RSD: < 30%<br>Recovery: 95−110 %	Testosterone: 0.57−4.25 µg/kg<br>Progesterone:.0.80−9.78 µg/kg	[32]
Estrone, 17β-estradiol, estriol and others EDCs	Chicken meat, fish and pond water	China	LLE, DLLME, derivatization (BCEC-Cl)	HPLC−MS	LOD: 0.03−0.07 µg/L<br>RSD: 3.1−5.4%<br>Recovery: 88−106%	Estriol: 3 .94 µg/L	[33]
Corticosterone, estrone, estriol, medroxyprogesterone, progesterone, 17β-estradiol, 17-α-hydroxyprogesterone, testosterone, and 19-nortestosterone	Fish tissue	China	DMAE, LLE	HPLC−MS/MS	LOD: 0.03−0.15 µg/kg<br>RSD: 3.3−5.6%<br>Recovery: 75−95%	Testosterone: 0.45 µg/kg<br>17α-Estradiol: 0.39−0.44 µg/kg<br>19-Nortestosterone: 0.30 µg/kg<br>17α-Hydroxyprogesterone: 0.26 µg/kg<br>Corticosterone: 0.57 µg/kg	[34]
Progesterone, testosterone, estrone, 17β-estradiol, 17α-ethinyl estradiol and others EDCs	Fish (sea catfish, croaker, marine gastropod mollusks)	Malaysia	QuEChERS	HPLC−MS/MS	LOD: 0.07−3.50 µg/kg<br>Recovery: 64−114%	17β-Estradiol: 0.14−0.91 µg/kg<br>Estrone: 0.04−0.38 µg/kg<br>Progesterone: 0.39−9.57 µg/kg<br>Testosterone: 0.16−0.7 µg/kg	[38]
Hexestrol, diethylstilbestrol, estrone, 17β-estradiol, 17α-ethinyl estradiol, estriol, testosterone, dihydrotestosterone, androstenedione, progesterone, norethindrone, levonorgestrel and pregnenolone	Fish and meat	Spain, Norway, Italy, Morocco, Greece, Portugal, Germany, USA	LLE, SPE derivatization (BSTFA−1% TMCS)	GC−MS	LOD: 0.0004−0,0150 µg/kg<br>RSD: 3.1−6.8 %<br>Recovery: 90−105%	Hexestrol: 0.020 µg/kg<br>Estrone: 0.060−0.970 µg/kg<br>17β-Estradiol: 0.170−0.550 µg/kg<br>Testosterone: 0.030−0.200 µg/kg<br>Dihydrotestosterone: 0.026−1.90 µg/kg<br>Androstenedione: 0.160−0.320 µg/kg<br>Progesterone: 0.0910.780 µg/kg<br>Levonorgestrel: 0.030−0.400 µg/kg<br>Pregnenolone: 0.380−0.810 µg/kg	This work

[a] AALLME air-assisted liquid−liquid microextraction; ASE accelerated solvent extraction; BCEC-Cl 2-(11H-benzo[a]carbazol-11-yl) ethyl carbonochloridate; BSTFA−1% TMCS *N,O-Bis*(trimethylsilyl)trifluoroacetamide containing 1% trimethylchlorosilane; DLLME dispersive liquid–liquid microextraction; DMAE dynamic microwave-assisted extraction; DMI dummy molecularly imprinted; EDCs endocrine disrupting compounds; FUSLE focused ultrasound solid–liquid extraction; GC–MS gas chromatography−mass spectrometry; HPLC– MS/MS high-performance liquid chromatography–tandem mass spectrometry; LC-MS/MS liquid chromatography−tandem mass spectrometry; LLE liquid−liquid extraction; LOD limit of detection; LOQ limit of quantification; MAE microwave assisted extraction; MSPD matrix solid-phase dispersion; MSPE magnetic solid phase extraction; MSTFA/NH_4I/DTE *N*-methyl-*N*-(trimethylsilyl)trifluoroacetamide/ammonium iodide/dithioerythritol; NH_2OCH_3-HCl *O*-methylhydroxylamine; QuEChERS Quick, Easy, Cheap, Effective, Rugged and Safe; RSD relative standard deviation; SPE solid-phase extraction; UAE ultrasonic assisted extraction; UHPLC−Orbitrap−HRMS ultra-high performance liquid chromatography−Orbitrap−high resolution mass spectrometry; UHPLC−MS/MS ultra-high performance liquid chromatography−tandem mass spectrometry; UPLC−MS/MS ultra-performance liquid chromatography−tandem mass spectrometry

Table S2. Variables influencing the sample pretreatment, solid-phase extraction/clean-up process and derivatization of hormones.

Variable	Optimum range (selected value)
Sample pretreatment[a]	
Volume of water-acetonitrile mixture 9:1 (v/v) (mL)	> 8 (10)
Centrifugation rate (rpm)	3500-5000 (4000 rpm)
Centrifugation time (min)	11-16 (12)
Centrifugation temperature (ºC)	0-10 (4 ºC)
Continuous solid-phase extraction/clean-up[b]	
Sample pH	6.5-7.5 (7)
Amount of Oasis HLB sorbent (mg)	75-85 (80)
Volume of acetone (eluent) (μL)	550-650 (600)
Flow rate of sample (mL/min)	4-6 (5)
Flow rate of air (mL/min)	4-6 (5)
Breakthrough volume (mL)	1-200 (10)
Derivatization [c]	
Percentage of TMCS (catalyst) in BSTFA[c]	1-5 (1)
Microwave oven power (W)	180-220 (200)
Irradiation time (min)	3.5-4.5 (4)

[a] To 2 g of meat and fish samples.
[b] These variables have been previously optimized [35].
[c] TMCS, trimethylchlorosilane; BSTFA, *N,O*-bis(trimethylsilyl)trifluoracetamide.

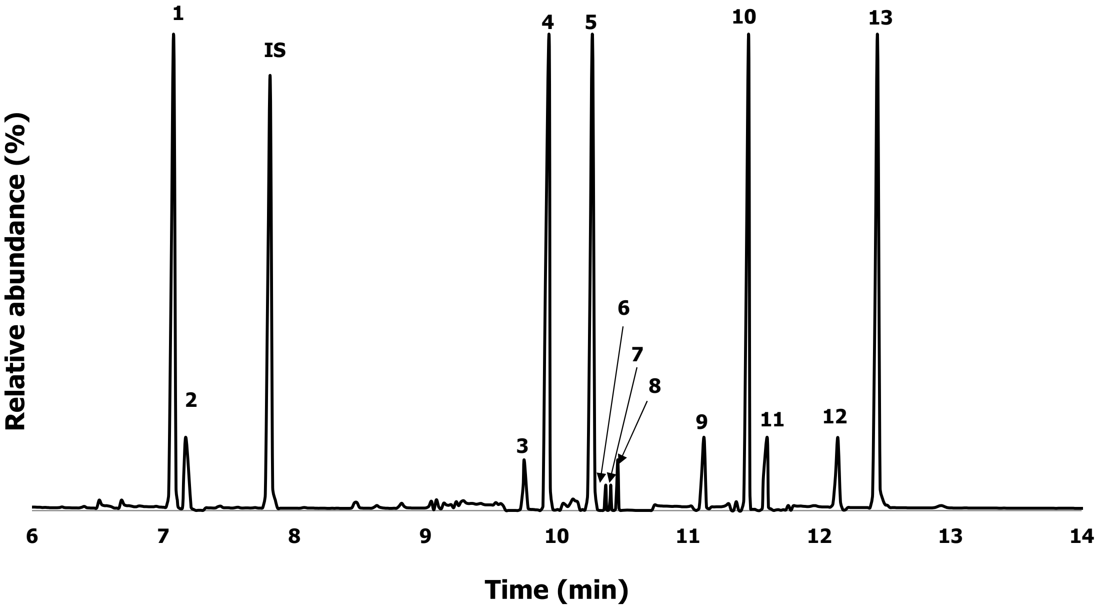

Figure S1. Typical chromatogram in the SIM mode for 1 g of chicken breast spiked with all hormones. Concentration: 250 ng/kg [(1) hexestrol, (4) estrone, (5) 17β-estradiol, (10) 17α-ethinylestradiol, (11) levonorgestrel, (12) progesterone, (13) estriol] and 500 ng/kg [(2) diethylstilbestrol, (3) dihydrotestosterone, (6) androstenedione, (7) norethindrone, (8) testosterone and (9) pregnenolone]. IS: internal standard.
